# Vitamin D levels and risk of delirium: A mendelian randomization study in the UK Biobank

**DOI:** 10.1212/WNL.0000000000007136

**Published:** 2019-03-19

**Authors:** Kirsty Bowman, Lindsay Jones, Luke C. Pilling, João Delgado, George A. Kuchel, Luigi Ferrucci, Richard H. Fortinsky, David Melzer

**Affiliations:** From the Epidemiology and Public Health Group (K.B., L.J., L.C.P., J.D., D.M.), University of Exeter Medical School, Royal Devon & Exeter Hospital, Exeter, UK; UConn Center on Aging (G.A.K., R.H.F., D.M.), University of Connecticut, Farmington, CT; and National Institute on Aging (L.F.), Baltimore, MD.

## Abstract

**Objective:**

To estimate effects of vitamin D levels on incident delirium hospital admissions using inherited genetic variants in mendelian randomization models, which minimize confounding and exclude reverse causation.

**Methods:**

Longitudinal analysis using the UK Biobank, community-based, volunteer cohort (2006–2010) with incident hospital-diagnosed delirium (ICD-10 F05) ascertained during ≤9.9 years of follow-up of hospitalization records (to early 2016). We included volunteers of European descent aged 60-plus years by end of follow-up. We used single-nucleotide polymorphisms previously shown to increase circulating vitamin D levels, and *APOE* variants. Cox competing models accounting for mortality were used.

**Results:**

Of 313,121 participants included, 544 were hospitalized with delirium during follow-up. Vitamin D variants were protective for incident delirium: hazard ratio = 0.74 per 10 nmol/L (95% confidence interval 0.62–0.87, *p* = 0.0004) increase in genetically instrumented vitamin D, with no evidence for pleiotropy (mendelian randomization–Egger *p* > 0.05). Participants with ≥1 *APOE* ε4 allele were more likely to develop delirium (e.g., ε4ε4 hazard ratio = 3.73, 95% confidence interval 2.68–5.21, *p* = 8.0 × 10^−15^ compared to ε3ε3), but there was no interaction with vitamin D variants.

**Conclusions and relevance:**

In a large community-based cohort, there is genetic evidence supporting a causal role for vitamin D levels in incident delirium. Trials of correction of low vitamin D levels in the prevention of delirium are needed.

Delirium is an acute decline in cognitive function, with a rapid onset of symptoms.^1^ It is relatively common in later life and is associated with substantially increased morbidity and mortality.^1,2^ Interest has grown in the potential role of vitamin D in protecting against cognitive decline. A recent meta-analysis reported that low vitamin D levels are associated with poorer cognition and accelerated cognitive decline,^3^ perhaps through vitamin D's anti-inflammatory properties.^4,5^ In observational studies, low levels of vitamin D have been associated with an increased likelihood of delirium in patients with hip fracture^6^ and for hospital-acquired, new-onset delirium.^7^ Genetically increased levels of vitamin D have been linked to reduced risk of Alzheimer disease in one study.^8^ However, evidence for the potential role of genetically increased vitamin D levels in reducing risk of incident delirium in large population-based studies is lacking.

Studies of outcomes in later life are susceptible to confounding and reverse causation, when associations with a risk factor are due to the effects of underlying pathology rather than being causal. Inherited genetic variants can provide stronger evidence for potential causal effect on an outcome—a technique known as mendelian randomization (MR).^9^

To apply genetic analyses, variants altering risk factor status are needed, which have their mode of action through the risk factor only.^9^ Here, we applied MR approaches to test vitamin D associations with incident delirium in an exceptionally large sample of older UK Biobank participants of European descent, in order to clarify the likely causal significance. We also accounted for *APOE* status.

## Methods

Between 2006 and 2010, UK Biobank recruited 503,325 community-based volunteers (aged 40–70 years) from across the United Kingdom.^10^ Genetic data were available on 488,377 UK Biobank participants, of whom 451,427 participants were identified as of European ancestry using self-report and genetics data.^11^ Given that 7.5% of the participants were from non-European ancestries and from diverse lineages, we focused on participants from European ancestry.

### Standard protocol approvals, registrations, and patient consents

Participants provided informed consent for data linkage to hospital inpatient admissions, cancer registrations, and death registrations. Ethical approval for the UK Biobank study was obtained from the North West Multi-Centre Research Ethics Committee. The current analysis was part of UK Biobank–approved project 14631 on aging well.

Delirium diagnosis was ascertained using ICD-10 code F05 in hospital discharge data. Participants who had a delirium episode before baseline interview were excluded from analyses (n = 51). Participants were eligible for inclusion if they reached the age of 60 within the follow-up period (up to February 16, 2016) (excluded n = 138,251). Participants with incident delirium had to be aged 60 years or older at diagnosis because incident cases before age 60 years are rare (excluded n = 3). One participant was excluded because they were missing an assessment date. This left 313,121 participants for the analysis.

Genotyping quality control and imputation were performed centrally by UK Biobank. Genetic variants rs429358 and rs7412 were used to define *APOE* haplotypes. Genetic variants for circulating 25(OH)D concentration (vitamin D) were extracted from 2 studies: 4 from Vimaleswaran et al.^12^ and 6 from a more recent report by Jiang et al.,^13^ which included a larger sample size but log-transformed the vitamin D levels (the 4 loci from Vimaleswaran are included in the 6 from Jiang) (only one variant from each locus was included, and only if the final meta-analysis *p* value was <5 × 10^−8^; all included variants had sufficient imputation quality [>0.4] and no deviation from Hardy-Weinberg equilibrium [*p* > 1 × 10^−6^] in the UK Biobank participants). We checked for consistency across both results and used the weights from Vimaleswaren to obtain the hazard ratio (HR) per nanomoles/liter of vitamin D. To confirm that the genetic risk score (GRS) was predictive for vitamin D, we used data from the InCHIANTI Study, a cohort study of aging in the Tuscany region of Italy in which both vitamin D and genetic variants were available.

The primary MR analysis methods were performed using R (v3.4.1) package “MendelianRandomization” (v0.2.2). Genetic variants were individually assessed for their association with incident delirium, and the natural log-transformed subhazard ratios (sHRs) were aligned with the vitamin D–raising allele and effect from the previously published studies and submitted to the “mr_input()” function. We used the penalized robust inverse-variance weighted (IVW) regression result as the primary analysis, and checked the penalized weighted median and penalized robust MR-Egger analyses for consistency in the effect estimate, and finally whether the MR-Egger intercept significantly differed from zero to test for potential pleiotropy (i.e., whether the effect of the single-nucleotide polymorphisms [SNPs] on delirium is via a pathway other than the risk factor under assessment^14^). The exponential of the IVW regression coefficient was derived (due to the earlier logging of the estimates for the MR analysis) to give the risk change (HR) per unit of vitamin D (nmol/L). The UK National Institute for Clinical Excellence concluded that “there is consensus that levels below 25 nmol/L (10 ng/ml) qualify as ‘deficient,’ but beyond this there is currently no standard definition of ‘optimal’ 25(OH)D levels. Some sources suggest that levels above 50 nmol/L (30 ng/ml) are ‘sufficient,’ while 70–80 nmol/L (28–32 ng/ml) is ‘optimal.’”^15^ We therefore chose to present the effect of genetically instrumented vitamin D levels per 10 nmol/L increase, as this is a clinically relevant level. To derive the risk of incident delirium per 10 nmol/L of vitamin D, we raised the HR to the power 10.

In secondary analyses of vitamin D, we created a GRS by summing the number of vitamin D-increasing alleles carried by each UK Biobank participant, weighted by the effect on circulating vitamin D levels using PLINK v1.9^16^; we used the 6 loci from Jiang et al.^13^ We confirmed that our vitamin D GRS was associated with vitamin D levels in blood assays from 1,161 participants from the InCHIANTI aging study, using a linear regression model adjusted for age, sex, and study site, with natural log-transformed vitamin D levels as the outcome (coefficient = 1.29, 95% confidence interval [CI] 0.80–1.78, *p* = 3 × 10^−7^).

Estimates of associations between the vitamin D variants and incident delirium were from Fine and Gray competing risks regression^17^ adjusted for age, sex, genotyping array, and principal components 1–5 of the genotyping data to account for population structure. Estimates of associations between *APOE* haplotype and incident delirium were adjusted for age, sex, genotyping array, and principal components 1–5. In sensitivity analyses, we investigated the effects of adjusting for self-reported time spent in the sun in the summer, excluding one of each pair of participants related to the third degree or closer (identified by KING kinship analysis^18^), adjusting for calcium GRS (n = 5 of 7 variants published in reference 19 as rs1550532 were not available in the UK Biobank version 2 genetic data release, and we excluded rs1570669 because it is located at the same locus as rs6013897 in the vitamin D score on chromosome 20), excluding participants with diagnosed dementia from analyses (ICD-10 codes F00, F01, F02, F03, and G30), investigating the effect of a rare variant in *CYP2R1* (rs117913124)^20^ with incident delirium, and rerunning the analysis using a noncompeting risk Cox model. To investigate whether the vitamin D-increasing variants may be acting via other pathways, we first took advantage of recent large pQTL (protein quantitative trait loci) studies to determine whether these loci are associated with proteins other than vitamin D^21,22^ and, second, the catalog of published genome-wide association studies.^23^ Stata v14.1 (StataCorp, College Station, TX) was used for competing risks regression.

### Data availability

Data are available from the UK Biobank after submitting an application (ukbiobank.ac.uk/register-apply/). The syntax for conducting the analysis is available on request.

## Results

Of 313,121 participants included in analyses, 544 had at least one incident episode of delirium recorded in hospital discharge data. The mean time to delirium diagnosis was 4.6 years (SD 1.6 years). Mean age at baseline for all the participants was 61.7 years (SD 4.7 years) and 53.8% of the sample were women (table 1). The mean age at delirium diagnosis was 71.6 years (SD 4.12 years), the minimum and maximum age was 60.0 and 77.8 years, respectively. The overall follow-up was up to 9.9 years. There were 11,669 deaths during the follow-up period (198 deaths after delirium). Within 1 year after the delirium diagnosis, 151 (27.8%) had died, and within 5 years, 196 (36.3%) had died.

**Table 1:**
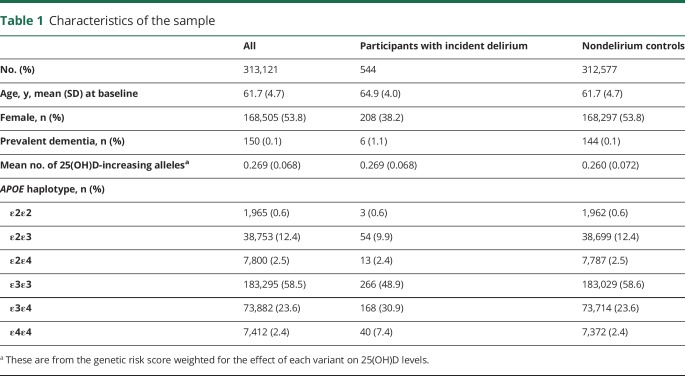
Characteristics of the sample

Vitamin D-increasing alleles were associated with decreased risk of delirium (figure, A and B and table 2): the IVW regression coefficient was −1.65 (95% CI −2.03 to −1.27, *p* = 3.5 × 10^−17^) (table 3). We observed consistent effect directions in the other MR methods (penalized weighted median coefficient = −1.71, 95% CI −3.04 to −0.38) (penalized robust MR-Egger coefficient = −1.90, 95% CI −2.61 to −1.19), and no significant pleiotropy, as tested by the MR-Egger method (*p* > 0.05).

**Figure:**
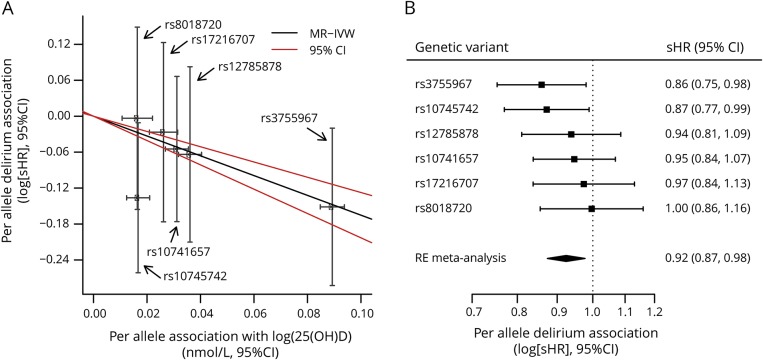
Vitamin D-increasing genetic variants are associated with reduced risk of incident delirium Jiang et al.^13^ (2018) found 6 genetic variants to affect circulating 25(OH)D (vitamin D) levels. (A) The effect of previously published per-allele effect of each variant on vitamin D levels is plotted on the x-axis, and the association of the same variant with incident delirium (logged hazard ratio) is plotted on the y-axis, with the mendelian randomization inverse-weighted (MR-IVW) regression line and 95% confidence intervals (CIs) also show. (B) The per-allele association with incident delirium is shown for each vitamin D–raising genetic variant, with a random-effects (RE) meta-analysis of the 6 variants also shown. sHR = subhazard ratio.

**Table 2:**
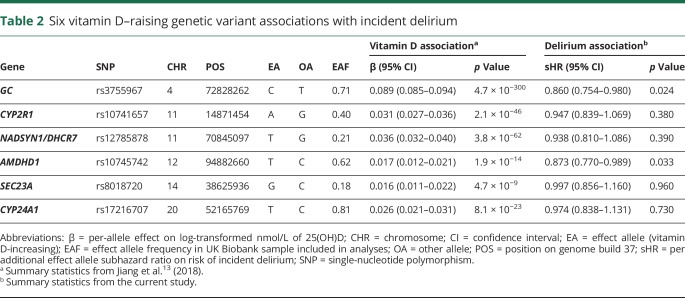
Six vitamin D–raising genetic variant associations with incident delirium

**Table 3:**
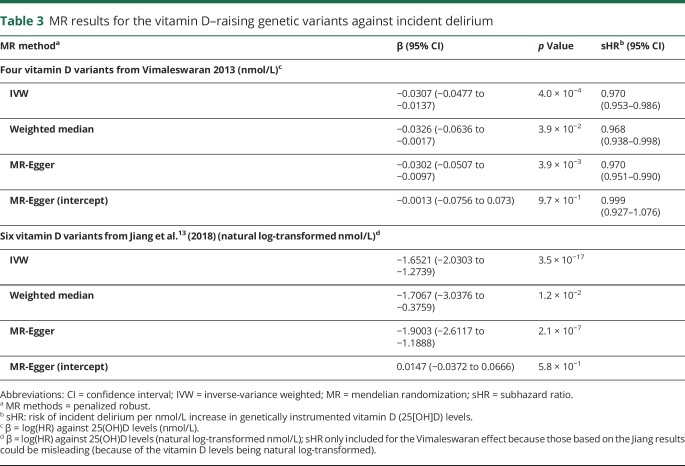
MR results for the vitamin D–raising genetic variants against incident delirium

Because the units from Jiang et al. are natural log-transformed vitamin D levels and therefore are not easily interpretable, we also applied MR methods to the 4 largest-effect variants published by Vimaleswaran et al.^12^ who provide the per-allele effect on each nanomole/liter of vitamin D levels. This relationship was consistent when we used 4 vitamin D-increasing alleles from Vimaleswaran et al.^12^ (table 3). The sHR for incident delirium per unit increase in genetically instrumented 25(OH)D levels (nmol/L) was 0.970 (the exponential of the penalized robust IVW regression coefficient −0.0307; table 3) (95% CI 0.953–0.986): incident delirium risk is therefore reduced by 3% per nmol/L increase in genetically instrumented vitamin D. The risk reduction per 10 nmol/L increase was 0.736 (95% CI 0.620–0.873), calculated by raising 0.970 to the power 10. We observed consistent effect directions in the other MR methods (penalized weighted median sHR = 0.968, 95% CI 0.938–0.998) (penalized robust MR-Egger sHR = 0.970, 95% CI 0.951–0.990), and no significant pleiotropy, as tested by the MR-Egger method (*p* > 0.05) (table 3). Using a GRS for vitamin D, we found a consistent association (sHR = 0.88 per SD of vitamin D GRS, 95% CI 0.81–0.96, *p* = 0.0044).

Participants with one or more *APOE* ε4 allele were at a substantial increased risk of incident delirium (table 4). Those homozygote for *APOE* ε4 (i.e., 2 ε4 copies present) had markedly increased risks (sHR = 3.73, 95% CI 2.68–5.21, *p* = 8.0 × 10^−15^); ε4 heterozygotes (i.e., ε3ε4) were at intermediate risk (sHR = 1.58, 95% CI 1.30–1.91, *p* = 3.8 × 10^−6^). The *APOE* associations were independent of the vitamin D genetic risks and were not substantively changed (table 4). There was no interaction with vitamin D variants.

**Table 4:**
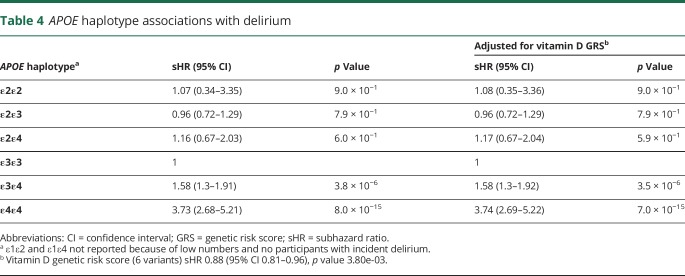
*APOE* haplotype associations with delirium

### Sensitivity analyses

To test the robustness of the vitamin D association with delirium, we adjusted for self-reported time spent outdoors in the summer; excluded one of each pair of participants related to the third degree or closer; and adjusted for a GRS of 5 known calcium level–altering variants that have not been reported to be associated with vitamin D levels. Results were not substantively changed (table 5) and the calcium-altering variants were not associated with delirium. Excluding 150 cases of dementia diagnosed before the assessment visit had no effect on the association between the vitamin D GRS, *APOE* genotype, and incident delirium (table 5). We tested the association between a low-frequency variant in *CYP2R1* (rs117913124, vitamin D–decreasing minor allele frequency 2.77% in the studied sample) identified in a separate study^19^ and incident delirium, and found there was no significant association (sHR 1.05, 95% CI 0.74–1.49, *p* = 0.78). However, because we had only 80% power to detect an effect of 1.6 (at α = 0.05, with 544 patients with delirium and 312,577 controls, using the genetic association study power calculator^24^), this variant may have a smaller effect on delirium that we were not powered to detect. In noncompeting risk models, the point estimates for the 6 vitamin D genetic variants with incident delirium were slightly different, although the overall conclusion remained the same (rs3755967 HR 0.86, 95% CI 0.76–0.98; rs10745742 HR 0.87, 95% CI 0.77–0.99; rs12785878 HR 0.94, 95% CI 0.81–1.08; rs10741657 HR 1.06, 95% CI 0.93–1.19; rs17216707 HR 1.03, 95% CI 0.88–1.19; rs8018720 HR 1.00, 95% CI 0.86–1.17). The MR analyses using results from noncompeting risk models were also consistent when using the 4 variants from Vimaleswaran: the HR for incident delirium per 10 nmol/L increase in genetically instrumented 25(OH)D levels was 0.737 (95% CI 0.620–0.872). Finally, we found that vitamin D-increasing loci (or proxies) are not associated with other protein levels in 2 recent large pQTL studies,^21,22^ although one of the 6 SNPs (rs17216707) is also associated with estimated glomerular filtration rate.^25^ Excluding this SNP from the MR analysis makes no meaningful difference (new penalized robust MR-IVW regression estimate using only 5 variants = −1.6399, 95% CI −1.887 to −1.392, *p* = 1.4 × 10^−38^).

**Table 5:**
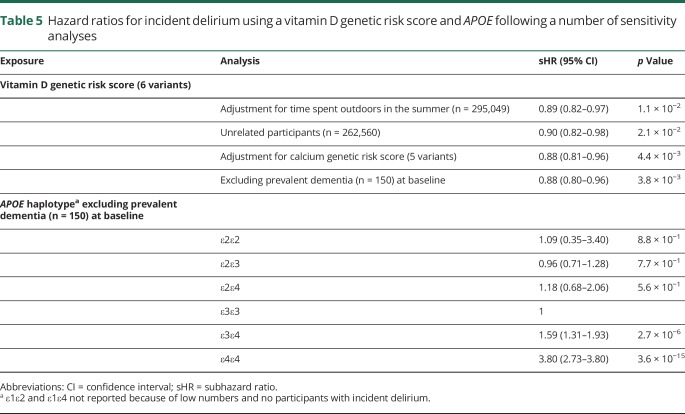
Hazard ratios for incident delirium using a vitamin D genetic risk score and *APOE* following a number of sensitivity analyses

## Discussion

In this large-scale prospective study, participants with genetically determined higher vitamin D levels had substantially lower risks of incident hospitalized episodes of delirium. Because MR analysis greatly reduces confounding and excludes reverse causation,^9,13^ our results suggest that interventional trials to increase vitamin D levels may be justified to reduce risk of delirium. In addition, participants carrying an *APOE* ε4 allele were at increased risk of incident delirium, likely contributing to the increased risk of dementia observed after an episode of delirium. However, the effect of vitamin D–related variants was not altered by *APOE* status.

The association between vitamin D and delirium is plausible as vitamin D receptors are distributed within the hippocampus, hypothalamus, cortex, and subcortex, and evidence suggests a variety of neurophysiologic and neuroprotective mechanisms.^26^ In addition, behavioral and attention disorders, and accelerated aging have been documented in vitamin D receptor knockout transgenic mice models.^27–29^ Furthermore, evidence indicates that vitamin D may modulate serotonin synthesis.^30^ Serotonin is involved in regulating a range of behaviors and brain function and has been indicted to have a role in delirium.^31^ Therefore, the effect of vitamin D on delirium may be in part attributable to its effect on serotonin synthesis.^30,31^ Low levels of vitamin D have been observationally associated with an increased likelihood of delirium in patients with hip fracture and for hospital-acquired, new-onset delirium.^6,7^

A number of studies have used MR approaches to estimate the effects of low vitamin D levels on cognitive outcomes. Of note, a recent analysis showed no association between vitamin D and global or memory cognition using an MR approach, although the vitamin D score (synthesis) was composed of 2 SNPs.^29^ Genetically increased levels of vitamin D (4 SNPs score), however, were associated with a reduced risk of Alzheimer disease at nominal statistical significance.^8^ Here, we use MR approaches to estimate the effects of vitamin D levels on risk of delirium, thereby minimizing confounding and excluding reverse causation. Although we cannot rule out pleiotropy (the vitamin D-increasing loci affect delirium risk via other mechanistic routes), we found no evidence in 2 large recent pQTL studies^21,22^ that these loci affect other protein levels, and excluding the one variant associated with estimated glomerular filtration rate in another genome-wide association study^25^ did not significantly change the result.

A recent meta-analysis reported that there was no association between *APOE* and delirium, although the number of delirium cases included was smaller than in the present study.^28^ Here, we showed carriers of an *APOE* ε4 allele were at an increased risk of delirium independent of a dementia diagnosis in our analyses, suggesting that *APOE* may constitute a shared mechanism between the 2 conditions. *APOE* ε4 status may therefore be a useful addition to estimating risk of developing delirium, especially in designing intervention trials.

There are a number of limitations with our study. First, serum vitamin D levels were not available for the UK Biobank participants and therefore the genetic variants could not be assessed concurrently with vitamin D levels and delirium. However, we confirmed that the genetic variants we studied were strongly associated with blood levels of vitamin D in the InCHIANTI Study (see methods). Second, MR analysis has important assumptions and limitations, in particular that the genetic instruments are only associated with the outcome via the exposure under examination, and the association between the exposure and outcome is linear; however, MR-Egger analysis provided no evidence of pleiotropy, and variants were not associated with covariates in our analyses. Finally, UK Biobank participants tended to be healthier than the UK general population at baseline, and therefore our number of delirium cases is lower than expected and estimates may be less applicable to frail older groups.^32^

In the data available, the vast majority of the participants had one delirium episode; 32 participants had ≥2 delirium episodes. Our analysis is based on the first delirium episode. Future studies could examine risk factors for recurrent events. The methods in this analysis were used to estimate the linear effect of vitamin D levels around the population average; future studies could examine the nonlinear effects of vitamin D and delirium.

In this study, genetic evidence suggests that higher vitamin D levels may be a substantial causal protective factor for incident delirium. Clinical studies are needed to confirm this. Delirium and dementia share a common risk factor in *APOE* genotype, which may contribute to the high rates of incident dementia observed after an episode of delirium.

## Glossary

CI: confidence interval
GRS: genetic risk score
HR: hazard ratio
ICD-10: *International Classification of Diseases, Tenth Revision*
IVW: inverse-variance weighted
MR: mendelian randomization
pQTL: protein quantitative trait loci
sHR: subhazard ratio
SNP: single-nucleotide polymorphism
